# The early diagnosis and pathogenic mechanisms of sepsis-related acute kidney injury

**DOI:** 10.1515/biol-2022-0700

**Published:** 2023-08-31

**Authors:** Wei Wei, Yibo Zhao, Yan Zhang, Songtao Shou, Heng Jin

**Affiliations:** Department of Emergency Medicine, Tianjin Medical University General Hospital, Tianjin 300052, P. R. China

**Keywords:** sepsis, acute kidney injury, biomarkers, pathogenesis

## Abstract

Sepsis is a syndrome caused by an imbalance in the inflammatory response of the body caused by an infection that leads to organ dysfunction, with the kidney being one of the most commonly affected organs. Sepsis-related acute kidney injury (SAKI) is strongly linked to increased mortality and poor clinical outcomes. Early diagnosis and treatment can significantly reduce patient mortality. On the other hand, the pathogenesis of SAKI is not fully understood, and early diagnosis of SAKI is a clinical challenge. Therefore, the current review describes biomarkers of acute kidney injury in sepsis and discusses the various pathogenic mechanisms involved in the progression of acute kidney injury in sepsis to develop new clinical treatment avenues.

## 1 Introduction

Sepsis is a serious disease caused by an unbalanced immune response to infections, which results in organ dysfunction. Sepsis can cause multiple organ dysfunction, including kidney dysfunction, leading to sepsis-related acute kidney injury (SAKI) [1]. Although antibiotics and organ function support have improved clinical outcomes, the mortality rate in patients with sepsis remains high [2]. Sepsis accounts for more than 50% of the causes of AKI, thus making it one of the most important causes [3]. This is further borne out by the results of the study of about 200,000 sepsis patients in the United States. The incidence of SAKI was 22%, with a case fatality rate of 38.2% [4]. SAKI has a complex and unique pathogenesis [5,6], and to date, it has been difficult to determine the exact onset time of SAKI in affected patients [7]. The occurrence of S-AKI is linked to negative outcomes. The prognosis of SAKI can be improved with early detection [8]. Therefore, the potential value of markers such as NAGL, kidney injury molecule 1 (KIM-1), insulin-like growth factor binding protein 7 (IGFBP7), and tissue inhibitor of metalloproteinase-2 (TIMP-2), among others, in the early diagnosis of SAKI is constantly being discovered [9,10]. Furthermore, compared to AKI caused by other causes, patients with S-AKI had a higher urinary microscopic score. Urine microscopy is only used to diagnose SAKI [11]. In this review, we identified biomarkers that may help in the early and rapid delineation of patients with SAKI and thus contribute to improved outcomes. We also further explore the complex pathogenesis of SAKI and how it affects disease progression.

## 2 Biomarkers for the early diagnosis of SAKI

**Figure 1:**
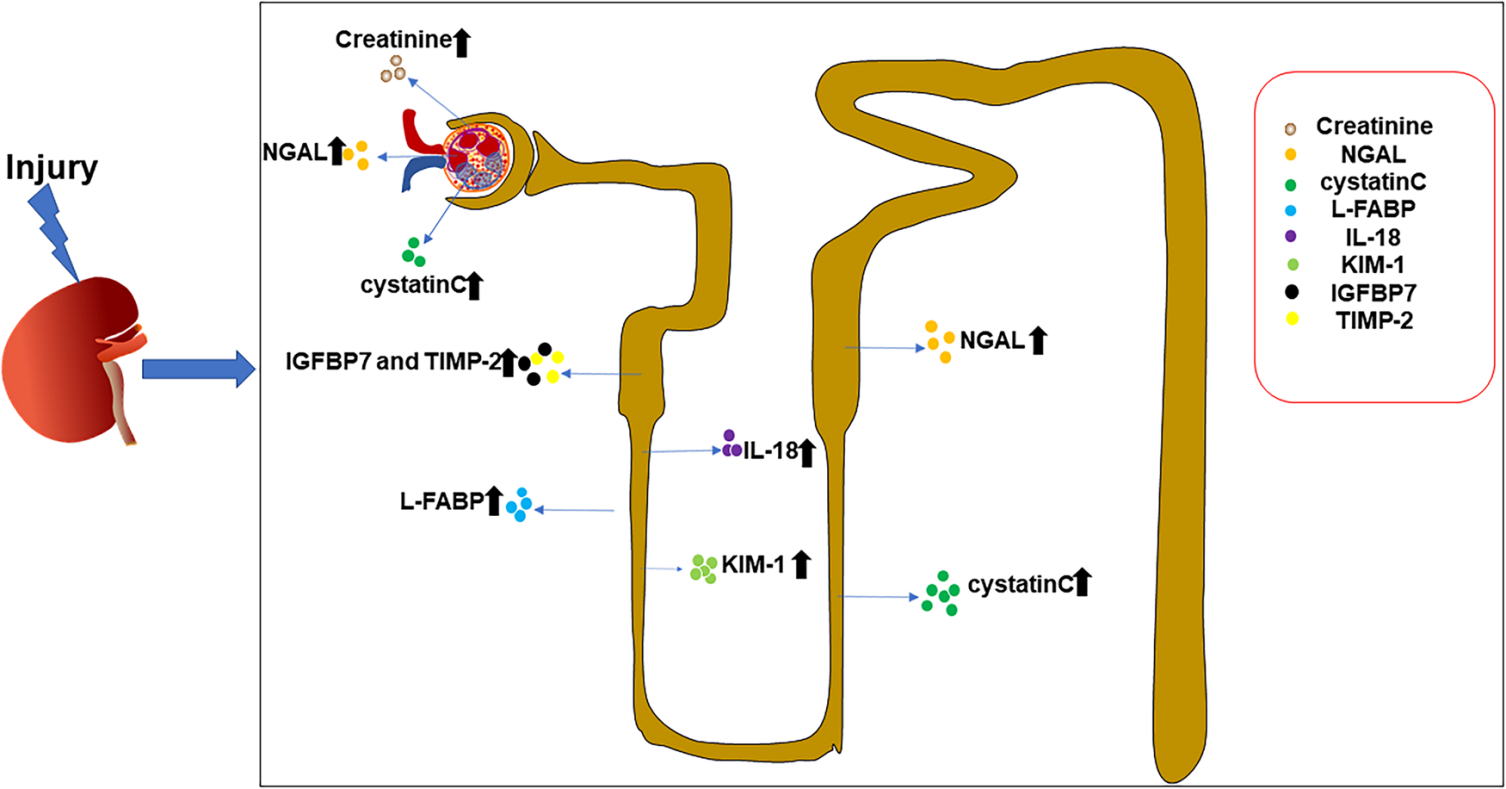
Biomarkers of SAKI at different sites. When the kidneys are injured, common biomarkers increase to varying degrees in different parts of the kidney.

Early detection of SAKI is critical for timely treatment and preventing further kidney damage. Serum creatinine and urea nitrogen measurements are still commonly used clinical indicators to diagnose AKI and to assess the severity and staging of AKI due to their convenience and low cost. On the other hand, serum creatinine and urea nitrogen levels are influenced by factors such as the patient’s age, nutritional metabolism level, and drug clearance rate. Only when the degree of renal injury reaches about 50% or even later in the course of renal injury, the serum creatinine level begins to increase, delaying diagnosis and early intervention [12,13,14]. Currently, many studies on the early diagnostic markers of different injury sites of SAKI (Figure 1) have been reported, and they have shown good sensitivity and specificity, which have early diagnostic value for SAKI (Table 1).

**Table 1:** Partial list of biomarkers for early detection of acute kidney injury in sepsis

Biomarker	Source of sample	Specificity	Sensitivity	AUC	Reference
NGAL	Urine	Urine; 0.84	Urine; 0.87	Urine; 0.92	[15]
	Serum	Plasma; 0.79	Plasma;0.83	Plasma; 0.87	
	Plasma	Plasma; 0.74	Urine; 0.80	Urine; 0.84	
KIM-1	Urine	0.74	0.84	0.62	[20]
CysC	Serum	0.84	0.82	0.96	[23]
IL-18	Urine	NR	NR	0.719	[27]
L-FABP	Urine	0.74	0.78	0.82	[29]
IGFBP7*TIMP-2	Urine	0.909	0.67	0.89	[32]

Notes: NR, not reported.

Abbreviations: AUC, area under curve; NGAL, neutrophil-associated lipid transporter protein; KIM-1, kidney injury molecule 1; CysC, cystatin C; IL-18, interleukin-18; L-FABP, liver-type fatty acid binding protein; IGFBP7*TIMP-2, insulin-like growth factor binding protein 7*tissue inhibitor of metalloproteinase-2.

### 2.1 Neutrophil-associated lipid transporter protein (NGAL)

In 1993, Kjeldsen discovered NGAL in cultured activated neutrophils and isolated it from peroxidase particles in neutrophils [15]. NGAL, also known as lipocalin 2 or 24p3, is a secreted protein with a relative molecular mass of 25,000. It widely exists in human tissues and organs including the uterus, liver, stomach, colon, breast, lungs, and kidneys, but it is rarely expressed under normal physiological conditions. When an organ suffers from inflammation, damage, or hemodynamic disorder, the expression of NGAL increases, particularly in the kidney [16]. In recent years, some large-scale basic and clinical studies [17,18] have confirmed that NGAL in urine, serum, and plasma has high sensitivity and specificity in the early diagnosis of SAKI. The sensitivity values of NGAL in urine, serum, and plasma were 0.87, 0.83, and 0.80; the specificity values were 0.84, 0.79, and 0.74; and the area under curves (AUC) were 0.9;2, 0.87, and 0.84. Urinary NGAL has a relatively high diagnostic value compared to serum and plasma NGAL. Therefore, NGAL is now recognized as a useful new marker for diagnosing SAKI.

### 2.2 KIM-1

KIM-1 is a transmembrane glycoprotein found on the epithelial cells of the proximal tubules of the kidney. KIM-1 expression is low in normal liver, kidney, spleen, and other organs but it increases significantly in renal proximal convoluted tubular epithelial cells (TECs) after kidney injury [19]. KIM-1 increased significantly faster than serum creatinine and urea nitrogen [20]. Tu et al. [21] found that after 24 h of admission to the ICU, the SCr level of SAKI patients began to increase. Urinary KIM-1, on the other hand, began to increase as early as 6 h later, peaked at 24 h, and remained increased for 48 h after admission to the ICU. Geng et al. [22] found that the sensitivity of urinary KIM-1 to diagnose SAKI was 0.74, the specificity was 0.84, and the AUC was 0.62. In summary, it is a better biomarker than serum SCr.

### 2.3 Cystatin C (CysC)

CysC is a protein with a molecular weight of 13 kDa. It is a cysteine protease inhibitor from the member’s superfamily of cysteine protease inhibitors. It is capable of being synthesized in all nucleated cells and is unaffected by objective factors such as age, gender, dietary structure, or muscle quality [23]. The amount of SCr and urea nitrogen in the body varies with muscle mass and diet [24]. In many studies for early diagnosis of SAKI, CysC was significantly better than SCr. Among them, Nejat et al. [25] compared the detection of functional changes in plasma CysC and plasma SCr in critically ill patients and discovered that the relative increase in plasma CysC occurred earlier than plasma SCr. Furthermore, Zhang et al. [26] demonstrated that the sensitivity and specificity of serum CysC in the early prediction of AKI were 0.84 and 0.82, respectively, with an AUC as high as 0.96. CysC levels in the blood are an effective and earlier predictor of decreased renal function. A recent clinical study by Al-Amodi et al. [27] found that TNF-α (−376 G/A) and cystatin could diagnose S-AKI and predict mortality in critically ill patients. The risk of S-AKI was significantly associated with sCysC, and the combination of sCysC and TNF-α (−376 G/A) outperformed sCysC alone in the diagnosis of S-AKI (AUC = 0.610, 0.838, respectively).

### 2.4 Interleukin-18 (IL-18)

IL-18 is a pro-inflammatory factor that belongs to the IL-1 cytokine superfamily. Activated monocytes, macrophages, dendritic cells, and NK cells are the most common sources of it [28]. Simultaneously, IL-18 is an inducer of IFN-γ. Increased levels of IL-18 in the body can cause the production of IFN-γ, TNF-α, and other cytokines, as well as the activation of immune-related cells, which can result in a variety of responses against bacteria, viruses, and fungi [29]. Several studies have found that an increase in IL-18 levels in urine can be detected in SAKI patients in 4–6 h, whereas changes in SCr usually take 2–3 days. In comparison to SCr, IL-18 promotes early prediction of the occurrence of SAKI [30]. Zhu and Shi [31] discovered that the IL-18 level of SAKI patients in ICU increased significantly at least 6 h earlier than SCr and that after 6 h, the area under the curve for IL-18 to diagnose SAKI was 0.719, which was better than 0.677 for SCr.

### 2.5 Liver-type fatty acid binding protein(L-FABP)

L-FABP is a 14 kDa small molecule protein produced primarily in the liver, expressed in various organs, and reabsorbed in the proximal tubules via glomerular filtration [32]. L-FABP is a member of a protein family that transports fatty acids and other lipophilic substances. It regulates lipid metabolism by combining with free fatty acids and transferring between the outer and inner cell membranes. In AKI caused by various causes of renal tubule ischemia and hypoxic stress, the excretion of L-FABP in urine is significantly increased [33]. A recent meta-analysis of 25 studies found that urine L-FABP had a sensitivity of 0.74 and a specificity of 0.78 in predicting AKI [34]. So, it is considered a valuable early biomarker of AKI.

### 2.6 IGFBP7 and TIMP-2

In 2013, IGFBP7 and TIMP-2 were identified as AKI biomarkers by Kashani et al. [35]. TIMP-2, a member of the tissue inhibitor of the metalloproteinases family and an endogenous inhibitor of metalloproteinase activity, has a molecular weight of about 24 kDa; IGFBP7 is a 29 kDa secretory protein; and they are all expressed and secreted by renal tubular cells [36]. TIMP-2 and IGFBP7 are produced and released by renal tubular cells in the early stages of injury caused by various injuries (such as sepsis, ischemia, and oxidative stress). TIMP-2 promotes the expression of p27, whereas IGFBP7 promotes the expression of p53 and p21. These proteins then inhibit the cyclin-dependent protein kinase complex (CyclD-CDK4 and CyclE-CDK2), causing transient G1 cell cycle arrest [35]. Nusshag et al. [37] recently discovered that [TIMP-2] × [IGFBP7] predicted the AUC of SAKI was 0.89, sensitivity was 0.909, and specificity was 0.67. Godi et al. [38] also demonstrated that combining [TIMP-2] × [IGFBP7] and PCT results may be an effective tool for identifying patients with SAKI and high-risk ICU short-term adverse outcomes.

## 3 Pathogenic mechanisms

Obviously, SAKI is usually caused by a complex mechanism rather than a single factor. Different pathogenesis causes kidney injury in different parts of the body. As previously stated, different markers are elevated in various parts of the kidney injury. It is unclear whether using the same markers to diagnose the injury of all kidney regions of SAKI improves the accuracy of early diagnosis and location of the injury [39]. Therefore, we will expand on the currently known pathogenesis of SAKI. The combination of different pathogenesis of SAKI and biomarker diagnosis at different sites of SAKI will aid in detecting clinical problems of SAKI early on and taking intervention measures to improve the prognosis of SAKI patients. Combining the various pathogenesis of SAKI and the diagnosis of biomarkers in different parts of SAKI will aid in identifying clinical problems of SAKI early on and taking intervention measures to improve the prognosis of SAKI patients.

### 3.1 Imbalance of inflammatory response

SAKI is a systemic inflammatory response syndrome caused by infections resulting in kidney dysfunction (Figure 1). Anti-inflammatory and pro-inflammatory factors disrupt the dynamic balance, resulting in a cascading inflammatory response storm, which has long been recognized as a key pathogenesis of the SAKI mechanism [6]. During SAKI, inflammatory mediators bind to membrane-bound pattern recognition receptors expressed by TECs, triggering a cascade of downstream signals that leads to the synthesis and release of pro-inflammatory factors. This resulted in renal tubule in necrosis and inflammatory cell infiltration in septic mice [5]. So far, a large number of studies have summarized the important role of inflammatory factors in AKI [40,41]. TNF, one of many inflammatory mediators and cytokines, can positively induce and promote the production of other inflammatory factors, resulting in the release of a large number of cytokines and producing a “waterfall effect” and is thus regarded as the key initiating factor for SAKI [42]. In clinical studies, Quinto et al. [43] found that severe SAKI patients with TNF removed by hemodialysis have lower mortality, which may be due to a weakening of the inflammatory response.

### 3.2 Cell death

**Figure 2:**
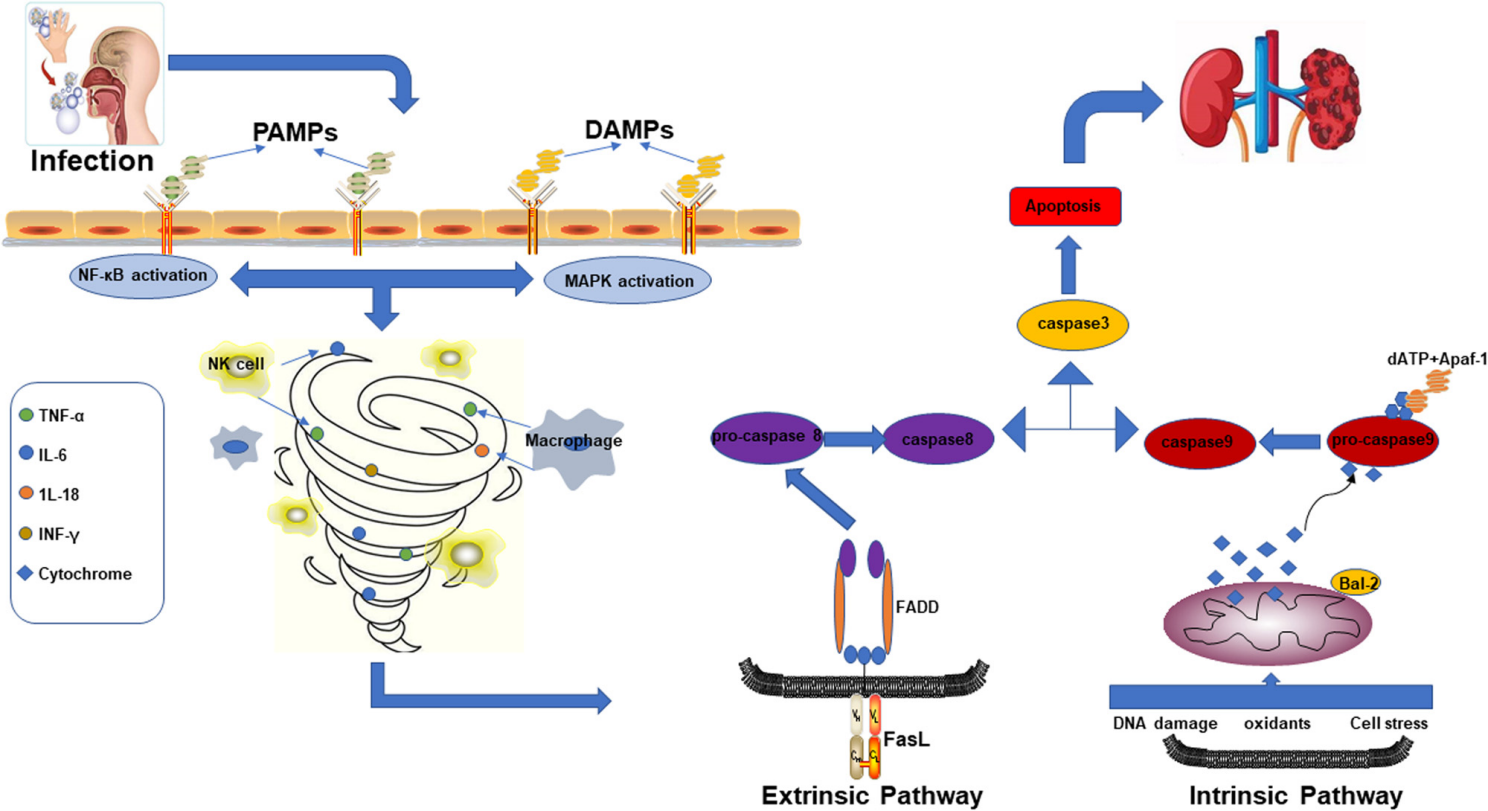
Inflammation disorder and apoptosis caused by severe infection. After human infection, pathogen-related molecular pattern PAMPs and disease-related molecular pattern DAMPs are recognized by toll-like receptors, activate NF-κB and MAPK inflammatory pathways, and induce immune cells (such as NK cells and macrophages) to secrete anti-inflammatory pro-inflammatory cytokines, which in turn trigger cell death.

Apoptosis refers to programmed cell death. Normal apoptosis is a response to the cell microenvironment that is essential for maintaining intracellular homeostasis [44]. A large number of renal tubular cells undergo inflammation, oxidative stress, and renal I/R injury in the early stages of SAKI’s kidney tissue, resulting in excessive renal tubular cell apoptosis. Multiple pathways, including internal (mitochondrial permeability transition pores, Bcl-2 family, cytochrome c, caspase-9) and external pathways, act together to cause apoptosis (FAS, FADD, caspase-8). On the one hand, in response to inflammation, cells undergo DNA damage, oxidative stress, and cellular stress, which can trigger endogenous cell apoptosis. In addition, cytochrome c released into the cytoplasm can interact with apoptosis-related factor 1 in the presence of dATP (Apaf-1) to form multimers, prompting caspase-9 to combine with it to form apoptotic bodies, and caspase-9 could activate caspase-3 after being activated. The binding of Fas and FasL, which are trimerized via the exogenous pathway, attracts the protein FADD with the same death domain in the cytoplasm to further synthesize with the inactive pro-caspase 8 (caspase-8) zymogen. The tropism cross-links and the caspase-8 molecule is then converted from a single-chain zymogen into an active double-chain protein, resulting in a cascade reaction, namely caspases [45], and the two pathways eventually act on caspase-3 to initiate the final apoptotic cascade. Caspase-3 is thus the “central link” in the process of cell apoptosis [46] (Figure 2). Yang et al. [47] confirmed *in vivo* that caspase-3 deficiency reduces microvascular endothelial apoptosis, renal tubular ischemia, and renal interstitial fibrosis in AKI mice. Furthermore, Ying et al. [48] discovered that drugs that inhibit caspase-3 expression could significantly protect SAKI mice and improve survival rates.

### 3.3 Cellular senescence

**Figure 3:**
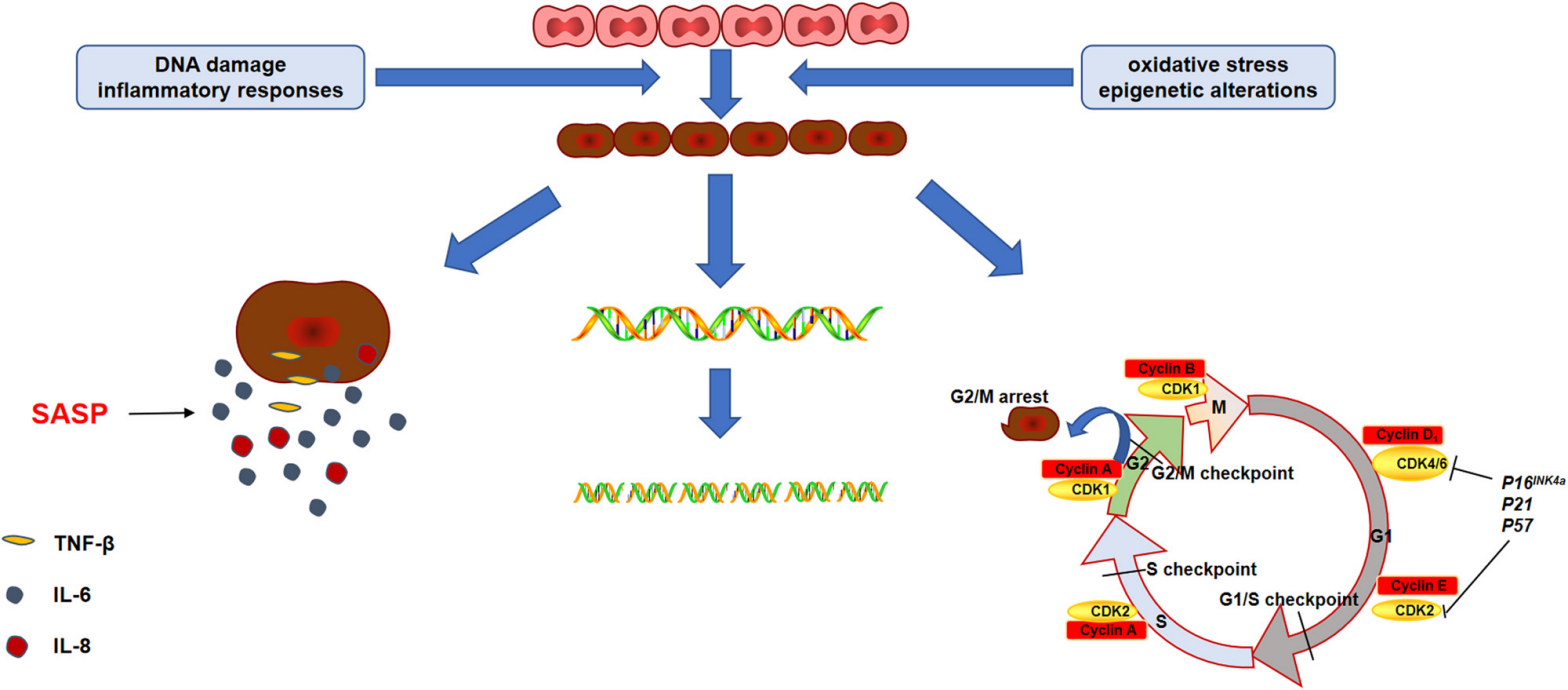
Typical features of senescence in renal TECs. Following SAKI, normal TECs undergo cellular senescence under conditions of DNA damage, inflammatory responses, oxidative stress, and epigenetic alterations.

Cellular senescence is a type of cell cycle arrest brought about DNA damage, inflammatory responses, oxidative stress, and epigenetic changes. DNA double-strand breaks, changes in cyclins, increased expression of cellular senescence-related proteins, and β-galactosidase are all hallmarks of cellular senescence. Simultaneously, senescent cells secrete senescence-associated secretory phenotype, which contains a variety of inflammatory factors such as interleukin-6 (IL-6) and transforming growth factor (TGF-β), etc. (Figure 3). Several studies in recent years have confirmed that cellular senescence plays a role in a variety of kidney diseases [49]. Jin et al. [50] confirmed that the TECs in mice with early AKI have undergone cellular senescence using AKI models induced by nephrotoxicity and ischemia-reperfusion injury. Furthermore, specifically knocking out the myeloid differentiation factor (Myd88) gene of TECs can reduce not only pro-inflammatory factors, interstitial infiltration, and fibrosis but also senescent cell aggregation and renal tubular damage, demonstrating that the TLR/Myd88/NF-κB pathway is an important pathway in mediating cellular senescence in TECs. Chen et al. [51] discovered that in the early stages of SAKI, Lipoxin A4 exerts anti-aging and anti-inflammatory effects via the PPAR-γ/NF-κB pathway, helping to improve renal function.

### 3.4 Mitochondrial dysfunction

The primary function of mitochondria is to generate energy. Because the kidney is a high-metabolic organ with a high energy demand, it is abundant in mitochondria [52]. When there is mitochondrial dysfunction, on the one hand, it will cause a decrease in ATP production and an increase in reactive oxygen species (ROS), resulting in a significant deterioration of renal function [53]. In contrast, ROS overload will cause a disruption mitochondrial electron transport chain and a change in membrane permeability. The release of cytochrome c promotes the occurrence of apoptosis and inflammatory response. Conversely, mitochondrial physiological autophagy and mitochondrial regeneration are critical in the recovery of renal function [54]. PGC-1α (PPARγ co-activator-1α) is a key regulator of mitochondrial regeneration [55]. When SAKI occurs, the kidney’s decreased expression of PGC-1α has a weakened protective effect on mitochondrial function, and the level of PGC-1α is directly related to the degree of renal injury [56]. Therefore, mitochondrial dysfunction is an important pathogenesis of SAKI, and mitochondrial function recovery is linked to renal function recovery.

### 3.5 Coagulation dysfunction

SAKI coagulation dysfunction is primarily caused by an imbalance between coagulation and anticoagulation in sepsis patients, endothelial injury and inflammatory response, fibrin mass production, and disruption of the fibrinolytic system [57]. SAKI frequently sets off a chain reaction of inflammatory storms, and the overexpression of inflammatory factors is related to coagulation dysfunction. Thrombin can stimulate inflammatory cytokines and the complement system, as well as platelet aggregation and white blood cell activation, resulting in diffuse microvascular injury. Antithrombin (AT) regulates thrombin but, in severe sepsis, the half-life of AT is shortened due to increased elastase destruction by activated neutrophils [58]. Furthermore, activated protein C is an endogenous anticoagulant with anti-inflammatory properties that is downregulated in sepsis [59]. Increased AT destruction due to thrombin dysregulation and decreased production of activated protein C will result in uncontrolled blood clotting. These effects may cause microvascular thrombosis and renal microcirculation injury, thereby aggravating the severity of SAKI. An ICU clinical study [60] discovered that, when compared to non-AKI patients, SAKI patients had significantly higher levels of APTT, PT, and D-dimers, and that APTT prolongation was independently related to patient mortality. APTT can be used to predict 30-day mortality independently.

### 3.6 Ischemia reperfusion injury

SAKI complicated by vascular endothelial injury, blood hypercoagulability, and overexpression of inflammatory factors can result in severe renal artery blood supply, insufficiency, resulting in ischemia and hypoxia of renal tissue. After a period of time, as the disease recovers, the vascular endothelial injury is repaired and hypercoagulable. After the blood supply to the kidney tissue is restored, thrombolysis, inflammation, and infection can be effectively controlled, and reperfusion injury can occur [61]. When the renal parenchymal cells are insufficiently perfused, the cellular mechanisms that maintain cell integrity are disrupted, leading to the initiation of the apoptosis pathway, and histological changes such as the disappearance of the brush border of the proximal tubules, the expansion of the distal tubules, and the formation of casts will further cause kidney obstruction. Intracellular calcium processing, xanthine signal transduction, and the formation of reactive oxidative substances are all involved in the process [62]. Furthermore, it is easy to produce a large number of oxygen free radicals and hydroxyl free radicals, during the process of restoring blood supply to the kidneys, which degrade polyunsaturated fatty acids in biofilms to cause lipid peroxidation and damage the structure and function of cells and mitochondrial membranes [63].

### 3.7 Autophagy

Autophagy is an intracellular decomposition process [64]. The body degrades organelles and proteins and forms autophagosomes when hunger or other pressures are stimulated to achieve cell homeostasis and organelle renewal [65]. Micro autophagy, macro autophagy, and molecular chaperone-mediated autophagy are the three main types of autophagy. The autophagy regulation mechanism is complex, with mTOR-dependent and mTOR-independent signaling pathways (AMPK, PI3K, Ras-MAPK, p53, PTEN) [66,67]. Under acute or chronic injury, moderate autophagy can play a renal protective role by regulating resident renal TECs [68]. On the one hand, renal injury causes an inflammatory cytokine storm, whereas autophagy has the ability to inhibit SAKI by targeting inflammatory bodies to regulate infection and prevent kidney disease [69]. Autophagy, on the other hand, can activate type I IFN reactions and promote IL-1B secretion [70]. Therefore, the pro-inflammatory and anti-inflammatory balance of autophagy can help prevent kidney disease caused by an overactive inflammatory response. An excessive inflammatory response is ultimately attributed to renal tubular epithelial injury and apoptosis, and autophagy plays an important role in renal tubular epithelial repair. Deng et al. found that SIRT1 alleviates SAKI via Beclin1 deacetylation-mediated autophagy activation, reduces creatinine urea nitrogen levels to some extent, and reduces renal tubular epithelial damage [71]. Similarly, Sun et al. confirmed in animal and cell experiments that p53 deacetylation promoted autophagy and alleviated SAKI [72].

## 4 Conclusion

Sepsis is a serious disease with a high morbidity and mortality rate. Multiple organ failure, particularly AKI frequently complicates sepsis. Patients with SAKI have a longer hospital stay and a higher risk of death in the hospital, which increases with the severity of AKI [73,74]. In clinical practice, the prognosis of SAKI patients is not encouraging, and the main treatments are dialysis and symptomatic and supportive care [75]. Individuals and countries bear a massive medical and economic burden as a result. Fortunately, the majority of the SAKI biomarkers discussed may be useful in early detection. Urine NGAL, plasma NGAL, CysC, IGFBP7, TIMP-2, and KIM-1 were the most promising. In addition to creatinine and urea nitrogen, urine NGAL, plasma NGAL, and KIM-1 are now frequently used as common indicators to assess kidney injury [39]. Furthermore, an increase in these markers may indicate the need for early RRT [76], with reference to a complex assessment of clinical parameters to reduce kidney injury and improve SAKI outcomes. Similarly, investigating pathogenesis may yield new ideas for treating SAKI. Recent research [77] has revealed that Klotho, as a protein expressed by renal cells, has anti-aging antioxidant properties. Klotho deficiency may worsen the severity of organ dysfunction in SAKI. Perhaps, this indicates that cellular senescence is one of the underlying pathogenesis of SAKI, pointing to a new treatment approach. Although progress in S-AKI pathogenesis has largely reached a new level in comparison to the past due to the promotion of *in vitro* and *in vivo* experiments, understanding of S-AKI pathogenesis still faces certain limitations [78]. Because of the critical condition of S-AKI patients, the risk of renal tissue biopsy far outweighs the benefit of pathological diagnosis, and data on the dynamic changes of S-AKI pathophysiology are lacking. Biopsy studies on patients who died from SAKI may provide some answers but the relevant data are limited due to medical ethics. To summarize, the ongoing advancement of diagnostic biomarkers and the pathogenesis of SAKI is beneficial for early targeted treatment and improvement of the kidney prognosis of SAKI. Following that, we may need to focus on the clinical application of biomarkers to better understand the role of multiple mechanisms inducing SAKI, as well as their relationship, and whether they can be converted into new therapeutic interventions.
